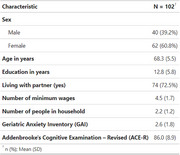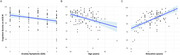# Association between anxiety symptoms and cognitive performance in cognitively healthy older adults in an upper‐middle‐income Latin American country

**DOI:** 10.1002/alz70857_099901

**Published:** 2025-12-24

**Authors:** Danilo Barroso de Sousa, Cecilia Patricia Popolin, Lucas Pelegrini Nogueira de Carvalho, Ari Alex Ramos, Márcia Regina Cominetti

**Affiliations:** ^1^ Federal University of São Carlos, São Carlos, São Paulo, Brazil

## Abstract

**Background:**

Anxiety and neurocognitive disorders are common clinical conditions among older adults, and evidence suggests that anxiety can disrupt cognitive performance. Hence, we sought to examine whether anxiety symptoms are associated with poorer cognitive performance in older adults from Brazil, an upper‐middle‐income Latin American country.

**Method:**

This cross‐sectional study enrolled 102 community‐dwelling older adults (60.8% females) aged 60 and over with no cognitive impairment. Participants were assessed using a sociodemographic questionnaire, the Addenbrooke's Cognitive Examination Revised (ACE‐R), the short form of the Geriatric Anxiety Inventory (GAI), and the Clinical Dementia Rating (CDR). A robust linear regression was implemented to analyze the potential associations between ACE‐R scores and the independent variables.

**Result:**

The fitted model revealed a significant main effect of anxiety symptoms (*F*_1, 94_ = 7.49; *p* adjusted = .020; Cohen's *f* = 0.28), indicating that older adults with higher scores on GAI exhibited poorer cognitive performance, independent of other variables in the model. Moreover, age (*F*_1, 94_ = 5.30; *p* adjusted = .047; *f* = 0.24) and educational achievement (*F*_1, 94_ = 23.63; *p* adjusted < .001; *f* = 0.50) were significantly associated with cognitive scores. In contrast, the main effects of sex (*F*_1, 94_ = 3.93; *p* adjusted = .080; *f* = 0.20), living with a partner (*F*_1, 94_ = 0.41; *p* adjusted = .526; *f* = 0.07), personal income (indexed as minimum wages) (*F*_1, 94_ = 1.46; *p* adjusted = .307; *f* = 0.12), and the number of people living in the household (*F*_1, 94_ = 0.53; *p* adjusted = .526; *f* = 0.07) did not reach significance.

**Conclusion:**

The current findings suggest that higher anxiety symptoms are independently associated with cognitive difficulties in later adulthood. This highlights the importance of addressing anxiety symptoms to mitigate their impact on cognitive outcomes and improve the quality of life at older ages.